# The Fabrication of Nanoimprinted P3HT Nanograting by Patterned ETFE Mold at Room Temperature and Its Application for Solar Cell

**DOI:** 10.1186/s11671-016-1481-y

**Published:** 2016-05-20

**Authors:** Guangzhu Ding, Kaixuan Wang, Xiaohui Li, Qing Chen, Zhijun Hu, Jieping Liu

**Affiliations:** College of Chemistry and Materials Science, Huaibei Normal University, Huaibei, 235000 China; Collaborative Innovation Center of Advanced Functional Composites of Anhui Province, Huaibei, 235000 China; Center for Soft Condensed Matter Physics and Interdisciplinary Research, Soochow University, Suzhou, 215123 China

**Keywords:** ETFE film, Nanoimprint lithography, P3HT nanograting, Solar cell

## Abstract

Nanoimprinting lithography (NIL) is investigated as a promising method to define nanostructure; however, finding a practical method to achieve large area patterning of conjugated polymer remains a challenge. We demonstrate here that a simple and cost-effective technique is proposed to fabricate the nanoimprinted P3HT nanograting by solvent-assisted room temperature NIL (SART-NIL) method with patterned ETFE film as mold. The patterned ETFE template is produced by embossing ETFE film into a patterned silicon master and is used as template to transfer nanogratings during the SART-NIL process. It indicates that highly reproducible and well-controlled P3HT nanograting film is obtained successfully with feature size of nanogratings ranging from 130 to 700 nm, due to the flexibility, stiffness, and low surface energy of ETFE mold. Moreover, the SART-NIL method using ETFE mold is able to fabricate nanogratings but not to induce the change of molecular orientation within conjugated polymer. The conducting ability of P3HT nanograting in the vertical direction is also not damaged after patterning. Finally, we further apply P3HT nanograting for the fabrication of active layer of OBHJ solar cell device, to investigate the morphology role presented by ETFE mold in device performance. The device performance of OBHJ solar cell is preferential to that of PBHJ device obviously.

**Electronic supplementary material:**

The online version of this article (doi:10.1186/s11671-016-1481-y) contains supplementary material, which is available to authorized users.

## Background

Conjugated polymers have been the focus of polymer and material science research in the past decades due to its excellent electricity, magnetic, and optic properties [1, 2]. Significant research efforts have been devoted to indicate that it is necessary to pattern the conjugated polymer film in pursuit of improving the performance of organic optoelectronic devices or requiring for special material processing [3]. Different patterning methods of conjugated polymer, either bottom-up or top-down techniques, have been employed to perform in the performance optimization and device fabrication, such as self-assembly, template, and irradiation patterning technique [4–6]. However, finding a practical method to achieve large-scale industrial patterning of conjugated polymer remains a challenge to now.

Among these techniques, nanoimprinting lithography (NIL) is investigated as a promising method to define nanostructures due to its high resolution, effective cost, and simple process [7, 8]. Thermal NIL technique is mainly able to transfer the patterns from the mold to resist materials by forced mechanical deformation and achieve patterned structure on the resist surface. Therefore, NIL is a typical template patterning method and has shown great potential for shaping functional soft materials into nanostructures. For example, thin films of semiconducting polymers [9–12], ferroelectric polymers [13, 14], and proteins [15] have recently been directly shaped to obtain desirous nanostructures with well-defined morphology. NIL is a cost-effective patterning method because a template can be used repeatedly without losing its reproducibility. Thus, like the typical template patterning technique, the control of nanoimprint mold is very significant in the fabrication of polymer pattern by NIL technique. Some traditional molds with high resolution over a large area, such as silicon mold or anodic aluminum oxide mold, are reported to apply; however, they are usually time-consuming, complicated process, simple fragility and crush or easy to be deformed, not to meet the demand of different commerce purpose. Thus, future template fabrication techniques which possess cost-effective and simple processing method will be the next pursuits significantly and a long way in this field will go for challenge.

The fluoro-polymer ethylene(tetrafluoroethylene) (ETFE) is a copolymer of ethylene and tetrafluoroethylene and has been attracting more and more attention due to its excellent property [16]. ETFE material possesses novel toughness, flexibility, and relatively high stiffness ability. Furthermore, ETFE film also bears a high melt point above 250 °C, which renders them useful in the thermal stability application. Thus, the copolymer is widely used in aerospace, nuclear utilization, and solar exploitation areas [17]. Correspondingly, ETFE films are intensively explored on the molecular parameters and microstructures [18, 19], thermal and crystallization behaviors [20, 21], and rheology property researches [16, 22]. Recently, Chen et al. [23] indicated that the flexible UV transparent ETFE mold could be fabricated by the hot embossing with the HSQ master template and further used to fabricate the OTFT device. The performance of the device was improved greatly, and the process was suitable for the roll-to-roll processing of flexible electronics. Barbero et al. [24] showed that ETFE thin film could be employed to fabricate the high-resolution nanoimprinting mold with a robust and reusable property. Densely packed nanostructures down to 12 nm into a wide range of various polymers were able to gain, and this high resolution is mainly dependent on the template’s mechanical stability and resistance to distortion at high pressure and temperature. The successful patterning techniques of a variety of technological important polymers have been proposed by employing the patterned ETFE film as mold; however, a comprehensive understanding of patterning by ETFE film mold remains unknown and is still a challenge, especially for the patterning formation of conjugated polymer by ETFE mold and its application.

In addition, room temperature NIL has been proposed to obtain desired patterning of conjugated polymer thin films because conjugated polymer material is inclined to easily oxidize and decompose at elevated temperatures, potentially detrimental to the property and application of conjugated polymer [5, 9, 25]. Therefore, it is also meaningful at room temperature to fabricate the nanoimprinting patterning of conjugated polymer by patterned ETFE template. Then, an ordered bulk heterojunction (OBHJ) morphology consisting of vertically aligned conjugated polymer nanostructure surrounded by the acceptor materials is important to gain high performance of solar cell device [25, 26]. NIL technique was investigated as a promising method to achieve this nanostructure morphology within the OBHJ solar cell and was able to enhance the device performance significantly [27–29]. Therefore, it is necessary to fabricate the pattern structure of conjugated polymer using patterned ETFE mold and further employ this technique to prepare the OBHJ solar cell for application, to investigate the morphology role presented by the ETFE mold in device performance.

In this paper, the patterned ETFE template was produced by embossing ETFE film into a patterned silicon master and solvent-assisted room temperature NIL (SART-NIL) with patterned ETFE mold was employed as a means to fabricate nanoimprinted nanopattern on the surface of poly(3-hexylthiophene) (P3HT) thin film. Then, the technique is applied to fabricate the active layer of OBHJ solar cell with P3HT as donor and PCBM as acceptor. We aim to report that a simple and cost-effective technique on the fabrication of nanoimprinted P3HT nanograting using patterned ETFE film as a mold is able to achieve the preparation and its application of patterned P3HT thin film at room temperature.

## Methods

Conjugated polymer P3HT (*M*_*w*_ 50 000 g mol^−1^; regioregularity 98 %) and PCBM (purity 99.5 %) were obtained from Rieke Metals Inc. and Solenne B. V. Co., respectively.

Simple and cost-effective technique on the fabrication of patterned ETFE mold was produced by the conventional thermal imprinting method. The patterned silicon master face was laid against the surface of ETFE thin film (100-μm thickness), and then exerted pressure (70 bar) at 240 °C and held for 15 min according to nanoimprinter system (Obducat, Eitre 3). Before releasing the pressure, the stacks were evacuated to solidify ETFE thin film and temperature was cooled down to room temperature (23 °C). After removing the silicon master, the patterned ETFE thin film was achieved successfully.

Solvent-assisted room temperature NIL (SART-NIL) with patterned ETFE mold was employed as a means to fabricate nanoimprinted nanopattern. The P3HT thin films were obtained by spin coating (1500 rpm) from chlorobenzene solution (20 mg ml^−1^) onto substrate. After spin coating for 10 s, the polymer films were immediately transferred to nanoimprinter system (Obducat, Eitre 3) and covered with patterned ETFE film. The nanoimprinting lithography process was performed under pressure (50 bar) at room temperature (23 °C) and held for 15 min. After the patterned ETFE thin film separated, the P3HT nanograting film was obtained.

The organic solar cells were fabricated with P3HT and PCBM as donor and acceptor materials, respectively. ITO-coated glass was washed with deionized water, ethanol, acetone, and isopropyl alcohol. After the glass was dried, PEDOT:PSS (about 30-nm thickness) was spin cast onto the ITO surface treated with ultraviolet ozone. Then, the whole substrates were annealed at 125 °C for 20 min in air. Nanostructured P3HT surfaces were prepared by the SART-NIL method described above. Then, PCBM in dichloromethane solution (10 mg ml^−1^) was spin coated (900 rpm) onto the top of patterned P3HT film under ambient atmosphere for 60 s. For the contrast devices, the planar bulk heterojunction (PBHJ) solar cell was also fabricated by spin coating PCBM onto the unimprinted P3HT thin film. In the end, the devices were completed by evaporating a LiF layer (0.8-nm thickness) protected by aluminum electrode (100-nm thickness) at a base pressure of 4 × 10^−4^ Pa. The effective photovoltaic area was 10 mm^2^.

The morphology of samples was investigated by scanning electron microscopy (SEM, Hitachi S-4800), operated voltage at 15 kV. Contact angle measurements were performed using a tensiometer (SL200C, Kino of American Company). Grazing incidence wide-angle X-ray diffraction (GIWAXD) measurements were performed at the BL14B1 beam line at the Shanghai Synchrotron Radiation Facility in China. The wavelength and the incident angle of the X-ray beam are 0.12398 nm and 0.18°, respectively. The platinum/iridium-coated cantilevers (0.2 N/m force constant from nanosensors) were employed for the conducting atomic force microscopy (C-AFM) (MFP-3D-SA, Asylum Research) measurements and the bias voltage between the ITO substrate and conducting cantilever (*V*_bias_) was 1.2 V under the atmosphere environment and at room temperature. UV absorption spectrum was performed using UV3600 spectrometer (Shimadzu) in the transmission geometry mode. Current-voltage characteristics of solar cells were measured under illumination of white light (100 mW cm^−2^) from a Hg-Xe lamp filtered by a Newport 81094 Air Mass Filter, using a Gwinstek SFG-1023 source meter. The EQE measurement was carried out with monochromatic light from Hg-Xe lamp (Newport 67005) and monochromator (Oriel, Cornerstone 260). The response was recorded as the voltage over a 50 Ω resistance, using a lock-in amplifier (Newport 70104 Merlin).

## Results and Discussion

**Scheme 1:**
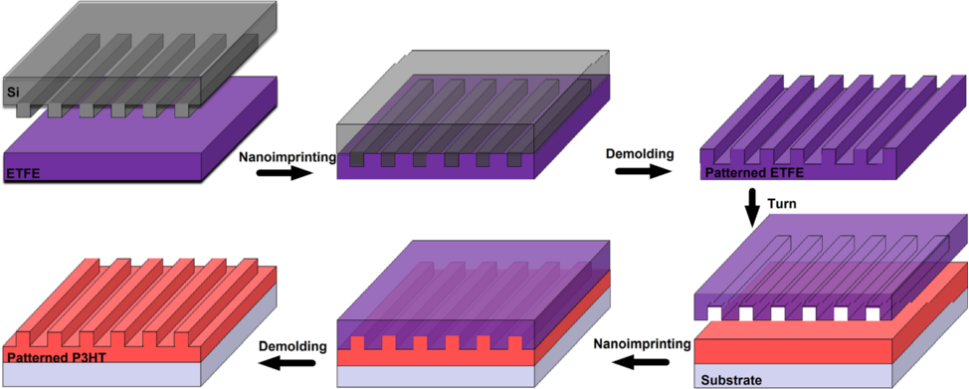
The fabrication process of patterned ETFE film mold and the process of nanoimprinted P3HT nanograting by SART-NIL method using patterned ETFE mold

We demonstrate here that the nanoimprinted P3HT grating film can be fabricated by a simple and cost-effective nanoimprinting method with patterned ETFE film mold at room temperature. The patterned ETFE template is produced by embossing ETFE film into a patterned silicon master and the SART-NIL method with patterned ETFE mold is employed as a means to fabricate nanoimprinted grating on the surface of P3HT thin film, as shown in Scheme 1. The fabrication process of patterned ETFE film consists of the preparation of desired ETFE thin film (thickness and smoothness) and the formation of ETFE nanostructure film replicated from patterned silicon master. The patterned silicon master is prepared in advance. The conventional thermal NIL method is employed to transfer the pattern on the surface of silicon to the ETFE thin film, and the details of NIL process are described in the “Methods” section. After removing the silicon master, the patterned ETFE thin film is achieved successfully. The thickness of ETFE thin film should be controlled carefully (about 100 μm or above), and it is difficult to sustain the mold itself if it is too thin. Since ETFE material possesses novel toughness, flexibility, and relatively high stiffness ability and bears a high melt point above 250 °C, therefore, higher temperature (240 °C) and pressure (70 bar) during the thermal NIL process enables the viscosity of ETFE film and is beneficial to the mobility of ETFE molecules, which contributes to fulfill the whole nanocavity of patterned silicon master. Next, it also provides a better conformal contact with the silicon master during nanoimprinting and it can be also released more easily from the silicon master once cooled down to room temperature. Finally, the nanopattern on the surface of ETFE film is retained from the silicon master precisely and is not able to change into swell or deform under ambient atmosphere and at room temperature, due to its stiffness. Then, due to little interaction between ETFE film and silicon material, there is no need for special surface treating of silicon master to facilitate master separation after nanoimprinting. In addition, it is noted here that typical silicon mold with high resolution over a large area is also able to fabricate the nanopattern of conjugated polymer, but they are usually time-consuming, complicated process, simple fragility and crush or easy to be deformed, not to meet the demand of various commerce purposes. However, the patterned ETFE mold is able to overcome the above weakness of silicon master during the NIL process. Therefore, it is significant to fabricate the patterned ETFE mold replicated from silicon master by this simple and practical process.

Next, SART-NIL method with patterned ETFE mold is employed as a means to fabricate nanoimprinted grating on the surface of P3HT thin film, as shown in Scheme 1. SART-NIL is a simple and cost-effective technique to fabricate the nanopattern of conjugated polymer at room temperature [30]. Room temperature NIL was proposed to obtain desired patterning of conjugated polymer films since conjugated polymer is incline to easily oxidize and decompose at elevated temperatures, potentially detrimental to the property and application of polymer [5, 9, 25]. The SART-NIL process consists of preparing P3HT thin films by spin coating for very short time and immediate nanoimprinting at room temperature. The residual solvent in the P3HT thin film resulting from short-time spin coating is able to lower the glass transition temperature and the viscosity of polymers during the NIL process, beneficial to the mobility of polymer molecule. It was reported that the high resolution was achieved due to the ETFE mold mechanical stability and resistance to distortion even at high pressures and high temperatures [24]. Therefore, under the effect of residual solvent, pressure, and the stiffness of the ETFE mold, the polymer P3HT is able to flow into the nanocavities of patterned ETFE mold during NIL process at room temperature. After removing the ETFE thin film, the nanoimprinting P3HT pattern on the surface of polymer thin film is obtained conveniently. In fact, a balance between stiffness and flexibility is present in the ETFE material. During the demolding, the ETFE mold is bent and gradually separated from the polymer surface using only a low force [24]. The ETFE mold does not break or deform and thus can be reused again for the employment to fabricate the P3HT nanostructure film for several times. Therefore, it is significant to employ this cost-effective and simple SART-NIL method using patterned ETFE film as a mold to fabricate the nanostructure of conjugated polymer.

**Fig. 1:**
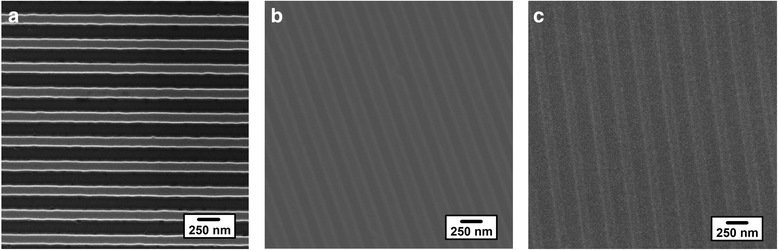
The top-down SEM images of silicon master, patterned ETFE mold, and P3HT nanograting film surface. **a** The top-down SEM image of patterned silicon master surface; **b** the top-down SEM image of patterned ETFE mold surface replicated from patterned silicon master surface; **c** the top-down SEM image of P3HT nanograting film replicated from ETFE mold using SART-NIL method. Scale bar = 250 nm

In order to estimate the application of cost-effective and simple SART-NIL method using patterned ETFE film as mold, the obtaining of highly reproducible and well-controlled P3HT nanogratings is very important. Examples of top-down SEM images of silicon master, patterned ETFE mold, and P3HT nanograting film surface are shown in Fig. 1, respectively, confirming that uniform arrays of P3HT nanogratings can be obtained by the SART-NIL method here. The patterned silicon master, shown in Fig. 1a, consists of regular nanogratings bearing ~150-nm-wide trenches and a ~280-nm center-to-center distance between the adjacent nanogratings (period). However, after the demolding of silicon master, the patterned ETFE gratings with ~150-nm width and ~280-nm period are obtained, as shown in Fig. 1b, which are faithfully transferred from the silicon master. Finally, according to SART-NIL method using patterned ETFE film, the P3HT nanograting shown in Fig. 1c contains uniform nanogratings bearing a width of ~130 nm and a period of ~280 nm. No collapses of P3HT nanogratings are observed in the film surface, and the width and period of obtained P3HT nanogratings precisely replicate the dimensions of ETFE mold. Therefore, it indicates that the nanoimprinted P3HT nanograting can be fabricated by this cost-effective and simple SART-NIL method using patterned ETFE film as mold.

**Fig. 2:**
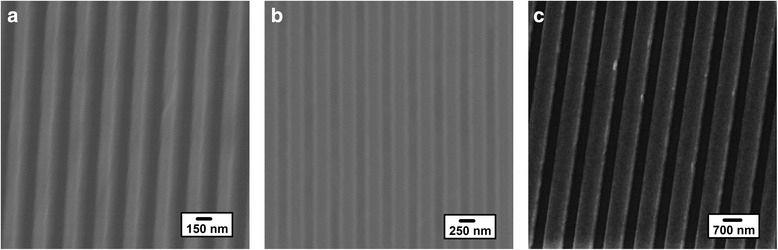
The top-down SEM images of P3HT nanograting film surface embossed from various patterned ETFE mold. **a** The top-down SEM image of P3HT film surface bearing nanograting of line width 150 nm, scale bar = 150 nm; **b** the top-down SEM image of P3HT film surface bearing nanograting of line width 250 nm, scale bar = 250 nm; **c** the top-down SEM image of P3HT film surface bearing nanograting of line width 700 nm, scale bar = 700 nm

Moreover, to further explore the effect of SART-NIL method using patterned ETFE film as mold on the preparation of polymer nanograting, we turn our attention to fabricate P3HT gratings with various feature sizes. Figure 2 shows the top-down SEM images of different P3HT nanograting film surfaces embossed from various patterned ETFE molds. The same preparation process is carried out as discussed above and we carefully control the fabrication conditions for films with various feature sizes. It indicates that perfect replication without deformation is obtained by patterned ETFE mold with line width of nanogratings ranging from 150 to 700 nm. It is noted that according to the fabrication process of nanoimprinted P3HT nanograting film, the highest aspect ratio of P3HT nanograting obtained (bearing a width of ~130 nm and a period of ~280 nm) is about 0.5, as shown in the Additional file 1. Here, we define that the aspect ratio is the ratio value of height to width within nanograting. Therefore, the height difference between the gratings and trenches is small and may lead to obscure contrast at the pattern edge within SEM images.

**Fig. 3:**
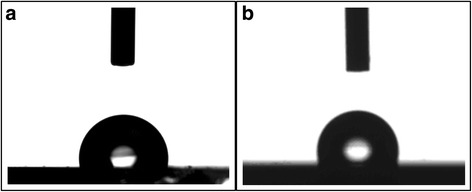
The telescopic images of water drop on the ETFE membrane surface before and after surface patterning by thermal NIL method. **a** The telescopic image of water drop on the ETFE membrane surface before thermal NIL process; **b** The telescopic images of water drop on the ETFE membrane surface after thermal NIL process

We note here that it is easy and simple to demold ETFE mold from the surface of conjugated polymer films without the nanostructure deformation. It can be revealed mainly by the flexibility and low surface energy of ETFE mold. Firstly, the achievement of high-resolution nanostructure by NIL technical needs to have an imprinting mold with mechanical stability and enough stiffness to prevent the nanostructure deformation during the NIL process. However, the effect of pressure often leads to the brittle fracture of mold with sufficient stiffness and the difficulty of mold demolding, such as silicon master. Thus, a balance between stiffness and flexibility of mold is beneficial to fabricate the steady and high-resolution nanopattern significantly. Fortunately, this balance is present in the ETFE mold. During demolding, the ETFE mold is bent and is able to separate gradually from polymer surface with only a small force. Thus, it is easy and simple to demold ETFE mold from the surface of conjugated polymer films, leaving the nanopattern and ETFE mold both complete without deformation and collapse. Secondly, an issue is that the mold often adheres to the polymer during imprinting, leading to the fracture of polymer pattern when demolding. To solve this problem, a lower surface energy material layer needs to be deposited onto the mold surface. However, the durability of deposited layer is limited and needs to be solved [31]. Here, the ETFE thin film is provided with low surface energy and it is revealed by the water contact angle as shown in Fig. 3. It shows the telescopic images of water drop on the ETFE membrane surface before and after surface patterning by thermal NIL method. It indicates that the water contact angle on the ETFE membrane surface is close to 108° and moreover the contact angle does not vary significantly before and after surface patterning by thermal NIL method. It can be inferred that a hydrophobic surface is present on the ETFE mold due to low surface energy and there is little interaction between ETFE mold surface and conjugated polymer P3HT, ensuring ETFE mold easily and simply to demold from the surface of conjugated polymer films without the nanostructure deformation. In addition, a hydrophobic surface is able to make the patterned ETFE film water insoluble and can be used at atmospheric environment. It will stay away from the damage of moisture greatly because the moisture enhances the degradation of P3HT-based organic electronics, detriment to device performance.

**Fig. 4:**
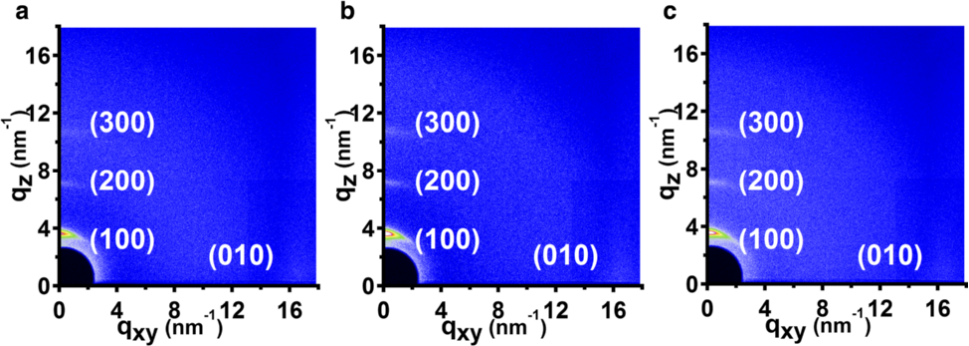
The two-dimensional (2D) GIWAXD images of P3HT thin film. **a** The 2D GIWAXD image of unimprinted P3HT thin film; **b** the 2D GIWAXD image of nanoimprinted P3HT thin film with grating direction parallel to the direction of incident X-rays; **c** the 2D GIWAXD image of nanoimprinted P3HT thin film with grating direction perpendicular to the direction of incident X-rays. The line width of P3HT nanograting is ~130 nm in the images **a** and **b**

SART-NIL method has been well explored to create nanostructures and to induce molecular orientation in conjugated polymer thin films. In our previous report [30], we demonstrated that large area poly(3-hexylthiophene) (P3HT) nanopillar arrays can be fabricated by a simple, cost-effective nanoimprinting method in a solvent-swollen plasticized state at room temperature, and this technology was able to induce face-on chain alignment in the P3HT nanopillars. Therefore, we also take (2D) GIWAXD image to investigate the P3HT chain alignment within nanograting film, as shown in Fig. 4. Similarly, we also define the diffraction vector *q*_xy_ and *q*_z_ pointing parallel and vertical to the substrate plane, and the peaks at *q* = 3.8 nm^−1^ and *q* = 16.8 nm^−1^ refer to the (100) plane and (010) plane reflections of P3HT crystal, respectively. It is noted here that the GIWAXD measurement are carried out with nanograting direction parallel and perpendicular to the direction of incident X-rays in order to investigate the molecular arrangement for clarity. It indicates that, as shown in Fig. 4a, (h00) and (010) reflections appear in the *q*_z_ and *q*_xy_, respectively, indicating that edge-on chain alignment dominates in the unimprinted P3HT thin films. After the patterning by SART-NIL method using ETFE film as mold, (h00) reflections in the *q*_z_ direction and (010) reflection in the *q*_xy_ direction are observed yet (Fig. 4b, c), indicating sole edge-on molecular orientation which is similar to the chain alignment of unimprinted P3HT thin film. Here, it is noted that the (010) reflection signals of samples are obscured generally within the 2D GIWAXD images. However, the (010) reflection signals of samples is present indeed in the *q*_xy_ direction, which can be indicated by the one-dimensional integrated curve stem from the Fig. 4a, b, c, as shown in the Additional file 2. The peaks at *q* = 16.8 nm^−1^ refer to the (010) plane reflections of P3HT crystal and can be investigated for the three samples. Furthermore, the signals of GIWAXD measurements do not change if the samples are set parallel or perpendicular to the direction of incident X-rays and it can be inferred that chain alignment displays a random distribution in the in-plane direction for nanoimprinted P3HT nanograting film. Therefore, it is suggested that the SART-NIL method using ETFE film as mold here is able to fabricate nanostructures but not to induce the change of molecular orientation within conjugated polymer thin film. It may be due to a different confinement dimension to induce chain alignment for P3HT nanograting during the SART-NIL process.

**Fig. 5:**
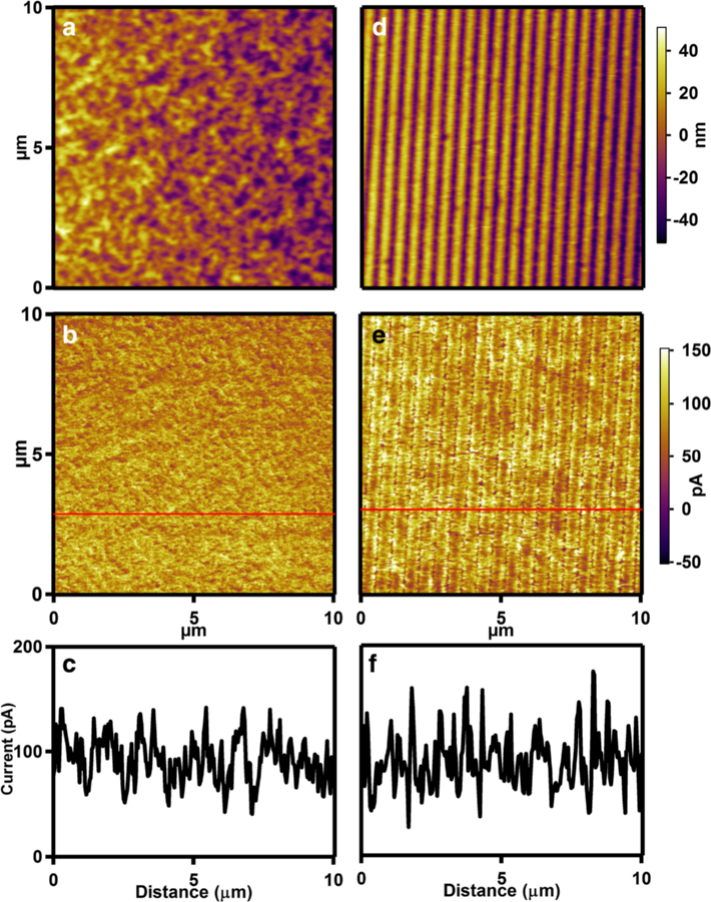
C-AFM height and current images of unimprinted and nanograting film. *Left column*: C-AFM height image (**a**), current image (**b**), and cross-sectional current graph (**c**) of unimprinted P3HT thin film. *Right column*: C-AFM height image (**d**), current image (**e**), and cross-sectional graph (**f**) of P3HT nanograting film. The *red lines* in graphs **b** and **e** show the directions of cross-sectional images **c** and **f**, respectively

Generally, the conducting atomic force microscopy (C-AFM) is used to assess the conducting ability of conjugated polymer film in the vertical direction. The platinum/iridium-coated cantilevers are employed for C-AFM measurements and the bias voltage between the ITO substrate and conducting cantilever (*V*_bias_) is applied to go through the thin film. Moreover, the morphology and conducting current of conjugated polymer film are obtained simultaneously during the C-AFM measurement. Here, the conducting performance of P3HT thin film is also investigated before and after patterning by employing C-AFM measurements, as shown in Fig. 5. A uniform film and regular P3HT nanograting arrays can be clearly observed from the unimprinted and nanograting films (Fig. 5a, d), respectively. Then, as for the current, a mean current of 100 pA is observed in the unimprinted film and distributed over the entire thin film. However, the nanoimprinted P3HT nanograting film gives a close current around 100 pA. It indicates that the conducting ability of unimprinted P3HT film is compared to the ability of nanoimprinted P3HT nanograting film in the vertical direction. The similar conducting ability between the unimprinting and nanograting films may be mainly determined by their unanimous edge-on molecular orientation as discussed above. In addition, it is noted that the current value of unimprinted film is not uniform and it mainly results from the difference of surface roughness within polymer film, which leads to the little change of current with position. Therefore, the conducting performance of P3HT thin film in the vertical direction is not damaged after patterning by SART-NIL method using ETFE film as mold.

**Scheme 2:**
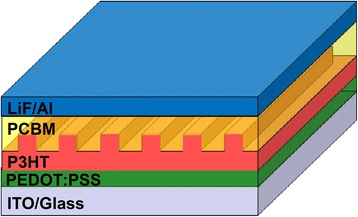
Schematic drawing for the structure of OBHJ solar cell device

To further apply the nanoimprinted P3HT nanograting film fabricated by the ETFE mold, we demonstrate here that the OBHJ solar cell is prepared using SART-NIL method, to investigate the morphology role presented by the ETFE mold in device performance. Scheme 2 shows the schematic drawing for the structure of OBHJ solar cell device. An ordered bulk heterojunction (OBHJ) morphology consisting of vertically aligned conjugated polymer nanostructure surrounded by the acceptor materials is an ideal structure to gain high performance of solar cell device [25]. NIL technique was investigated as a promising method to achieve this nanostructure morphology [27]. Here, P3HT and PCBM were used as donor and acceptor materials, respectively. P3HT nanograting film was obtained on the PEDOT:PSS-coated ITO/glass substrate, by using SART-NIL method with patterned ETFE mold. Subsequently, PCBM was spin coated onto the surface of P3HT nanograting arrays under an ambient atmosphere. LiF and the electrode were finally deposited onto the PCBM surface and then the fabrication of OBHJ solar cell was completed successfully. As we have known, in order to gain vertically aligned conjugated polymer nanostructure surrounded by the acceptor materials, the preparation of highly reproducible and well-controlled P3HT nanograting array is very important. However, we have successfully obtained the nanoimprinted P3HT nanograting film with uniformity and regularity as discussed above, to ensure the completion of OBHJ active layer desired. In addition, dichloromethane is chosen as a solvent of PCBM material because it dissolves PCBM well but not P3HT material; therefore, the depositing PCBM layer process would not destroy the P3HT nanogratings and is able to ensure the nanostructure formation of active layer for OBHJ solar cell. For the contrast devices, the planar bulk heterojunction (PBHJ) solar cell was also fabricated by spin coating PCBM onto the unimprinted P3HT thin film under the same processing condition. In all, it indicates that OBHJ solar cell is able to be fabricated and assisted by this simple and cost-effective SART-NIL method using patterned ETFE mold.

**Fig. 6:**
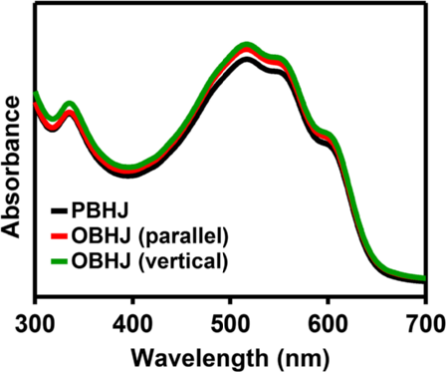
The UV-vis absorbance spectra of PBHJ and OBHJ films. Parallel and vertical are referred as measurements performed with nanograting line direction parallel and perpendicular to the direction of incident light. The line width of P3HT nanograting is ~130 nm within the OBHJ structure

Before, to investigate the performance of PBHJ and OBHJ active layers of solar cell device, the UV-vis absorption spectra of OBHJ and PBHJ films are recorded, as shown in Fig. 6, to obtain the effect of P3HT nanograting arrays fabricated by the SART-NIL method with ETFE mold on the optical absorption. The absorption peak centered at around 333 nm refers to typical absorption of PCBM and the peak locations do not vary for the three films obviously, indicating that the dimension of PCBM domains do not change largely [27, 28]. Then, the peaks at around 515, 550 (P3HT π–π^*^ transitions), and 601 nm (P3HT inter-chain π–π interaction) are the typical absorption peaks of the ordered arrangement polymer P3HT to a certain extent [27, 28]. It indicates that the absorption intensity of OBHJ film is enhanced after the pattern process with ETFE mold and higher than the intensity of PBHJ film. Due to no variation of the material amount and chain alignment of polymer (discussed above) after the NIL process, the enhancement of the UV-vis absorption intensity may be attributed to light trapping [28]. The periodic nanograting is able to enhance light trapping and thus increase the optical absorption by increasing the optical pass length. It is noted that, compared to PBHJ, the enhancement of UV-vis absorption intensity of OBHJ structure is limited. In fact, the whole UV-vis absorption process is complicated and the height of P3HT nanograting obtained (the biggest is about 70~80 nm) is also limited. In addition, the nanometer scale of P3HT nanograting obtained (bearing a width of ~130 nm and a period of ~280 nm) is finite yet. Thus, light trapping and the increasing of the optical pass length are limited relatively. Therefore, the absorptions only have very small difference. Here, we aim to report the fabrication of nanoimprinted P3HT nanograting by patterned ETFE mold at room temperature and its application for solar cell. The result indicates that the periodic nanostructure fabricated by NIL method is able to enhance the UV-vis absorption intensity and it is not our goal to promote the UV-vis absorption intensity only. Moreover, there is no obvious alteration of the absorption intensity for OBHJ films between the nanograting line directions parallel and normal to incident light direction. It further shows that the optical absorption of OBHJ nanostructure film displays a random distribution in the in-plane direction for unimprinted and nanoimprinted P3HT thin film. Therefore, it is suggested that the fabrication of OBHJ nanostructure using SART-NIL method with ETFE mold is able to promote the UV-vis absorption intensity, beneficial to the solar cell performance.

**Fig. 7:**
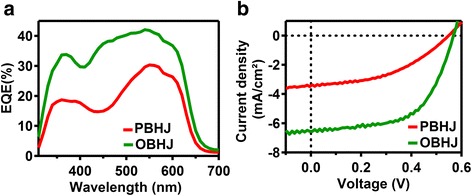
Device performance characteristics of solar cell. **a** EQE curves and **b** *J-V* curves of solar cells based on the PBHJ film and OBHJ active layers bearing P3HT nanogratings. The line width of P3HT nanograting is ~130 nm within the OBHJ solar cell device

Then, we studied here how these nanoimprinted P3HT nanogratings within OBHJ structure impact the performance of solar cell device. The photovoltaic properties of active layers based on OBHJ and PBHJ for comparison were evaluated by fabricating solar cell with a conventional device structure as shown in Scheme 2. The photovoltaic responses of solar cells were obtained by measuring EQE curve and current density versus voltage (J–V) curve under illumination, as shown in Fig. 7. The corresponding parameters are given in Table 1. All the measurements were performed under an ambient atmosphere and at room temperature.

**Table 1:** The device performance parameters of solar cells under the illumination

Active layer	V_OC_ [V]	FF	Jsc [mA cm^-2^]	PCE [%]
PBHJ	0.55	0.44	3.49	0.83
OBHJ	0.56	0.58	6.55	2.18

The whole curves exhibit a broad response covering 300–700-nm wavelength, as shown in Fig. 7a. It indicates that the EQE spectra of the devices show a similar shape to their absorption spectra of active layer used in the device, showing both components (P3HT and PCBM) contributing to the photocurrent. Moreover, the EQE curve of OBHJ solar cell is obviously enhanced compared to that of PBHJ solar cell. As discussed above, the molecular orientation and vertical conducting property of nanograting P3HT film are compared to unimprinted film. Therefore, the enhancement of EQE spectra of the devices may be due to optical absorption enhancement, interfacial area increase and bi-continuous pathway within device structure.

The devices show similar open circuit voltage values (0.55 and 0.56 V), indicating that current generation from exciton dissociation at the interfaces between P3HT nanograting arrays and PCBM occurs in the both devices. In contrast to the similar open circuit voltage values, the PSCs based on the OBHJ film show significantly improved Jsc (from 3.49 to 6.55 mA cm^−2^) due to the formation of bi-continuous transportation pathways (P3HT nanograting arrays and filled PCBM) by the SART-NIL technique with ETFE mold. Considering about the mechanism of bulk heterojunction solar cell, the improvement of circuit of solar cell device based on OBHJ film may be determined by many effects. Firstly, the fabrication of nanoimprinted P3HT nanograting can be ensured by this cost-effective and simple SART-NIL method using patterned ETFE film as mold. Uniform P3HT nanograting arrays in the OBHJ film can effectively raise the interfacial area and thus efficiently increase the exciton dissociation efficiency, leading to the Jsc improvement. Then, the enhanced light absorption intensity of P3HT nanograting arrays based on OBHJ film is able to absorb more photons from sunlight and further contributes to the enhancement of Jsc. Finally, compared to the device based on PBHJ film, the exciton within the device based on OBHJ film is able to more easily reach the interface between P3HT and PCBM due to the shorter pathway of exciton diffusion and the higher efficiency of exciton transportation, leading to facilitate the photocurrent generation. In addition, the improvement of exciton dissociation efficiency due to the increase of interfacial area is also able to reduce the exciton recombination rate during exciton dissociation process and thus it is certain to improve the FF value of device based on OBHJ active layer. In all, the whole device performance of OBHJ solar cell is preferential to that of PBHJ device obviously, especially in the Jsc, FF, and PCE. Therefore, it confirms that the SART-NIL method using patterned ETFE film as mold is an effective technique to fabricate an ideal active layer bearing bi-continuous pathways and significantly improve the solar cell performance.

It is noted that we aim here to report a simple and cost-effective technique on the fabrication of nanoimprinted P3HT nanograting film by SART-NIL method with patterned ETFE film as mold and its application for active layer of OBHJ solar cell device. The efficiency of OBHJ solar cell device here does not show even higher value anticipated (here 2.18 %); however, it is not our desire to gain the absolute value of solar cell efficiency. The low efficiency of solar cell based on OBHJ active layer film may be limited by many factors of device fabrication process, such as the feature size of nanograting structure or under an ambient atmosphere.

## Conclusions

In all, in this paper, we report a simple and cost-effective technique on the fabrication of nanoimprinted P3HT nanograting film by SART-NIL method with patterned ETFE film as mold and its application for active layer of OBHJ solar cell device. The patterned ETFE template is produced by embossing ETFE film into a patterned silicon master and SART-NIL method with patterned ETFE film as mold is employed as a means to fabricate nanoimprinted grating on the surface of P3HT thin film. It indicates that the highly reproducible and well-controlled P3HT nanograting film is obtained successfully with feature size of nanogratings ranging from 130 to 700 nm, due to the flexibility, stiffness, and low surface energy of patterned ETFE mold. Moreover, it is suggested that the SART-NIL method using ETFE film as mold is able to fabricate nanostructures but not to induce the change of molecular orientation within the conjugated polymer. The conducting performance of P3HT thin film in the vertical direction is also not damaged after patterning. Finally, we further apply the nanoimprinted P3HT nanograting film for the fabrication of OBHJ solar cell using SART-NIL method, to investigate the morphology role presented by the ETFE mold in device performance. P3HT and PCBM are used as donor and acceptor materials, respectively. It indicates that the whole device performance of OBHJ solar cell is enhanced obviously, especially in the Jsc, FF, and PCE.

## Additional Files

Additional file 1: The cross-section SEM image of P3HT nanograting film bearing a width of ~130 nm and a period of ~280 nm. According to the fabrication process of nanoimprinted P3HT nanograting film, the highest aspect ratio of P3HT nanograting obtained (bearing a width of ~130 nm and a period of ~280 nm) is about 0.5. Here, we define the aspect ratio is the ratio value of height (L) to width (W) within nanograting. (DOC 186 kb) Additional file 2: One-dimensional GIWAXD curves in the *q*_xy_ direction. Curves are integrated from Fig. 4a, b, c. Parallel and vertical are referred as measurements performed with nanograting line direction parallel and perpendicular to the direction of incident X-rays. It indicates that the (010) reflection signals of samples is present indeed in the *q*_xy_ direction, which can be indicated by the one-dimensional integrated curve stem from the Fig. 4a, b, c. The peaks at *q* = 16.8 nm^−1^ refer to the (010) plane reflections of P3HT crystal and can be investigated for the three samples. (DOC 45 kb)
