# Supplementary material for: The Fabrication of Nanoimprinted P3HT Nanograting by Patterned ETFE Mold at Room Temperature and Its Application for Solar Cell
Source: Nanoscale Res Lett. 2016 May 20;11:258. doi: 10.1186/s11671-016-1481-y (PMC4875018; doi:10.1186/s11671-016-1481-y)
Supplement: Additional file 1: — The cross-section SEM image of P3HT nanograting film bearing a width of ~130 nm and a period of ~280 nm. According to the fabrication process of nanoimprinted P3HT nanograting film, the highest aspect ratio of P3HT nanograting obtained (bearing a width of ~130 nm and a period of ~280 nm) is about 0.5. Here, we define the aspect ratio is the ratio value of height (L) to width (W) within nanograting. (DOC 186 kb) [file 11671_2016_1481_MOESM1_ESM.doc]

**Additional file 1**

**The cross-section SEM image of P3HT nanograting film bearing a width of ~ 130 nm and a period of ~ 280 nm.**

According to the fabrication process of nanoimprinted P3HT nanograting film, the highest aspect ratio of P3HT nanograting obtained (bearing a width of ~ 130 nm and a period of ~ 280 nm) is about 0.5. Here we define the aspect ratio is the ratio value of height (L) to width (W) within nanograting.
