# Supplementary material for: The Fabrication of Nanoimprinted P3HT Nanograting by Patterned ETFE Mold at Room Temperature and Its Application for Solar Cell
Source: Nanoscale Res Lett. 2016 May 20;11:258. doi: 10.1186/s11671-016-1481-y (PMC4875018; doi:10.1186/s11671-016-1481-y)
Supplement: Additional file 2: — One-dimensional GIWAXD curves in the q xy direction. Curves are integrated from Fig. 4a, b, c. Parallel and vertical are referred as measurements performed with nanograting line direction parallel and perpendicular to the direction of incident X-rays. It indicates that the (010) reflection signals of samples is present indeed in the q xy direction, which can be indicated by the one-dimensional integrated curve stem from the Fig. 4a, b, c. The peaks at q = 16.8 nm−1 refer to the (010) plane reflections of P3HT crystal and can be investigated for the three samples. (DOC 45 kb) [file 11671_2016_1481_MOESM2_ESM.doc]

**Additional file 2**

**One dimensional GIWAXD curves in the** **q_xy_ direction. Curves are integrated from Figures 4a, 4b and 4c. Parallel and vertical are referred as measurements performed with nanograting line direction parallel and perpendicular to the direction of incident X-rays.**

It indicates that the (010) reflection signals of samples is present indeed in the q_xy_ direction, which can be indicated by the one dimensional integrated curves stem from the Figures 4a, 4b and 4c. The peaks at q=16.8 nm^-1^ refer to the (010) plane reflections of P3HT crystal and can be investigated for the three samples.
